# The anti-staphylococcal activity (planktonic and biofilm) of *Cnestis ferruginea* is due to benzoquinone, the oxidation product of hydroquinone

**DOI:** 10.3389/fcimb.2024.1494589

**Published:** 2025-01-17

**Authors:** Sujogya Kumar Panda, Michelle Reynders, Purity N. Kipanga, Walter Luyten

**Affiliations:** ^1^ Department of Biology, Katholieke Universiteit (KU) Leuven, Leuven, Belgium; ^2^ Centre for Biotechnology, Siksha ‘O’ Anusandhan (Deemed to be University), Bhubaneswar, India

**Keywords:** *Cnestis ferruginea*, benzoquinone, hydroquinone, oxidation, biofilm, *S. aureus*

## Abstract

**Introduction:**

*Cnestis ferruginea* is used frequently in African traditional medicine for treating infectious diseases. Previous bioassay-guided purification has identified hydroquinone as the major bio-active compound in the aforementioned plant, responsible for its antibacterial activity against *Staphylococcus aureus*. While the phenol hydroquinone can be directly extracted from the plant, it may undergo (reversible) oxidation under mild conditions to yield benzoquinone, a compound with known antimicrobial activity against *i.a. S. aureus*.

**Methods:**

We, examined whether hydroquinone or its oxidation product, benzoquinone, is the active compound against bacteria such as *S. aureus*. To achieve this we performed broth microdilution (planktonic) and biofilm activity tests against two different strains of *S. aureus*. The inhibitory concentrations (IC_50_) of benzoquinone and hydroquinone under various circumstances were compared, assessing their stability, and examining their effectiveness against two strains of *S. aureus* (Rosenbach and USA 300) in both planktonic and biofilm environments.

**Results:**

Benzoquinone demonstrated antibacterial activity against *S. aureus* Rosenbach and USA 300 with IC_50_ of 6.90 ± 2.30 mM and 7.72 ± 2.73 mM, respectively, while the corresponding values for hydroquinone were 15.63 ± 2.62 mM and 19.21 ± 4.84 mM, respectively. However, when oxidation was prevented by the addition of antioxidants such as ascorbic acid or glutathione, hydroquinone lost its antibacterial property, while benzoquinone retained activity. Comparing conditions in which hydroquinone could convert into benzoquinone against conditions in which this conversion was inhibited, showed that hydroquinone alone did not inhibit bacterial growth of *S. aureus*, while benzoquinone alone did.

**Discussion:**

These results prove that the oxidation product benzoquinone is responsible for the antimicrobial activity previously ascribed to hydroquinone.

## 1 Introduction

A current problem in modern medicine is the antimicrobial resistance crisis. Due to, among other causes, misuse, and overuse of antimicrobials, a rapidly increasing number of microbial strains is no longer susceptible to antimicrobials. This has caused infections that were previously relatively easily cured to become a threat again (Ventola, 2015). According to the European Centre for Disease prevention and Control (ECDC) antibiotic resistance causes approximately 25.000 deaths annually in Europe alone. Furthermore, resistance carries a high economic cost because patients infected with resistant microbes show a longer duration of illness than those infected with non-resistant microbes. This in turn lengthens the interval during which the infection can be spread to others. The cost of resistant infections, according to the ECDC, ranks up to an estimated 1.5 billion euros annually (European Centre for Disease Prevention and Control, 2010). An important hospital-acquired microbial species with a history of antimicrobial resistance is the bacterium *Staphylococcus aureus*, a Gram-positive coccal bacterium which can be a commensal, and is present in the nasal cavity of around 30 percent of humans. However, the bacterium can also act as an opportunistic pathogen and is one of the most common causes of infection in healthcare facilities. Afflictions range from small infections such as sinusitis and epidermal abscesses to life-threatening infections such as pneumonia, meningitis, and sepsis (Tong et al., 2015). As a consequence, new treatments for resistant infections are sorely needed. Amongst possible new treatments are novel antimicrobials, novel antimicrobial combinations and alternative methods such as vaccines (Tagliabue and Rappuoli, 2018) and bacteriophage therapy (Golkar et al., 2014).

The use of medicinal plants to treat a wide range of illnesses and ailments is an ancient practice that is still used in most parts of the world today and can provide a starting point for discovering new bio-active compounds. *Cnestis ferruginea* is a plant native to Africa, more specifically to the region from Senegal to Cameroon, and is used as traditional medicine against a wide range of conditions. For instance the stem is used to treat throat infections, the fruit is employed as a treatment for oral infections and the root gets used as a laxative (Ha, 2017). Kouakou et al. (2019) used bioassay-guided purification to isolate antimicrobial compounds from the leaves of the plant. Bioassays on *S. aureus* resulted in the isolation of an active compound, which was identified using LC-MS and NMR as hydroquinone, a phenol. The authors concluded that this is the major compound responsible for the antibacterial activity against *S. aureus*. We noticed, however, that the color of bacterial growth media changed during the broth microdilution assay in wells with hydroquinone. This suggested that hydroquinone underwent changes during incubation. One such possible change is oxidation to benzoquinone, a colored reaction product. Phenols are aromatic structures consisting of a phenyl ring bonded to a hydroxyl group. They can be readily oxidized to a quinone, which has a cyclic dione structure. Hydroquinone carries a second hydroxyl group in para position on the benzene ring. It can easily be oxidized to benzoquinone. Both the phenol and quinone chemical classes contain compounds with antimicrobial effects. Quinones especially have shown great potential as antimicrobials due to their tendency to interact with nucleophilic amino acids, causing protein disruption, and their potential to supply free radicals (Cowan, 1999). However, quinones also have cytotoxic and carcinogenic effects. Bolton and Dunlap have proposed that the cytotoxicity of quinones has an inverse U-shaped curve, whereby toxicity is a function of reactivity on one hand and dose on the other. To avoid host toxicity when using quinones as an anti-microbial treatment, it is advised to lower the dose as it will decrease reactivity (Bolton and Dunlap, 2017). The purpose of the present investigation is to examine the antibacterial activity of benzoquinone, an oxidized derivative of hydroquinone; the latter was previously isolated from *C. ferruginea* leaves. Specifically, the study aims to examine whether benzoquinone is the primary compound responsible for the antibacterial effects against *S. aureus*. This will be accomplished by comparing the inhibitory concentrations (IC_50_) of benzoquinone and hydroquinone under various circumstances, assessing their stability, and examining their effectiveness against two strains of *S. aureus* (Rosenbach and USA 300) in both planktonic and biofilm environments.

## 2 Materials and methods

### 2.1 Bacterial strains, growth media, and chemicals

Most chemicals were purchased from Sigma-Aldrich, Belgium, and used without additional purification unless otherwise specified. Resazurin salt and Dimethyl suphoxide (DMSO) were purchased from Acros Organics (Geel, Belgium), and CHEM-LAB NV (Zedelgem, Belgium), respectively. Sterile deionized water was produced by a water purification system (Milli-Q Reagent Water System, MA, United States). All chemical stock solutions were aliquoted and stored at -20°C.

Tryptic soy broth (TSB) without dextrose was purchased from Life Technologies (Bacto™ Tryptic Soy Broth without Dextrose), and 100 mL was prepared according to the manufacturer’s instructions. After autoclaving, 500 μL of sterile 40% D-glucose (in water) was added. Cells from overnight cultures were washed with phosphate-buffered saline (PBS) 1x buffer, and resuspended in TSB medium. PBS 10x stock (8% NaCl, 0.2% KCl, 1.44% Na_2_HPO_4,_ 0.24% KH_2_PO_4_) was prepared and filter-sterilized. Then, PBS 1x buffer was obtained by diluting 10 mL of PBS 10x with 90 mL sterile MilliQ water.

The following *S. aureus* strains were used for antibacterial tests: *S. aureus* subsp. *aureus* Rosenbach 65385 and *S. aureus* strain USA300. The latter is a methicillin-resistant bacterium that easily forms biofilms. All strains were stored in 20% glycerol in cryovials at −80°C. Material from the frozen stock was streaked out on tryptic soy agar (TSA) plates, which were then incubated at 37°C for 24 hours before being stored at 4°C.

### 2.2 Antimicrobial activity of hydroquinone and benzoquinone on planktonic cells

The antimicrobial activity was determined using a broth microdilution method, as described earlier (Panda et al., 2017). A colony from a Petri plate was taken, suspended in 3 mL TSB liquid medium, and incubated in a shaker incubator at 37°C and 100 rpm for 18-24 hours. These overnight cultures were vortexed, and 1 mL was transferred to a 1.5 mL sterile tube. The tube was then centrifuged at 7000 rpm for 2 minutes at ambient temperature. The supernatant was discarded and 1 mL PBS was added. The pellet was resuspended, and a second centrifugation at 7000 rpm for 2 minutes at ambient temperature was performed. The supernatant was again discarded, and the pellet was resuspended in 1 mL TSB medium. The optical density (OD) was measured in a spectrophotometer at 620 nm.

The two-fold dilution series prepared for hydroquinone and benzoquinone quantification (see 2.4), were also used to test the antimicrobial activity of the compounds. Ten µL of the two-fold dilution series of the desired compound was added to 190 µL TSB medium with bacterial cells (either *S. aureus* Rosenbach or *S. aureus* USA300) in a 96–well flat-bottom polystyrene microtiter plate (COSTAR). This was then followed by an incubation (stationary) at 37°C for 24 hours, after which the plate was read by a Multiskan FC Microplate Photometer at 620 nm to obtain the OD of each well. Controls used in the experiment were wells filled with ten μL DMSO and 190 μL TSB medium with bacterial cells, as well as wells filled with 10 μL of either hydroquinone or benzoquinone and 190 μL TSB medium without bacterial cells. Ciprofloxacin was used as a positive control throughout all experiments (Panda et al., 2019; Van Puyvelde et al., 2021).

The percentage of inhibition was calculated with the following formula:


$$\text{Inhibition percentage }=\left(100\;-\;\frac{\text{test reading }\left(\text{OD}\right)\;-\text{ coloration reading }\left(\text{OD}\right)}{\text{average of solvent control readings }\left(\text{OD}\right)}\right)x\;100$$

Test readings are the OD of wells where both microbial cells and hydroquinone or benzoquinone were present. coloration readings are the ODs of wells where hydroquinone or benzoquinone was present in TSB medium without any bacterial cells; this correction was necessary due to the red color of benzoquinone (and to a certain extent hydroquinone). Solvent control readings provide the OD of wells with microbial cells and DMSO but no hydroquinone or benzoquinone. The entire experiment was performed under reduced light conditions (in evening, without switching on light in laminar flow hood, keeping plates wrapped in aluminum foil whenever possible) because hydroquinone and benzoquinone are light-sensitive.

### 2.3 Antimicrobial activity of hydroquinone and benzoquinone on biofilms formed by *S. aureus*

For biofilm tests with the USA 300 strain, the resazurin procedure as described in Kipanga et al. (2020) was followed with slight modifications. A standardized inoculum was prepared by adjusting the OD of the starter culture to 0.1 (10^6^ cells). Then, 190 μL of this standardized inoculum was placed in each well of a 96-well flat-bottom plate and incubated for 90 minutes. Wells were rinsed with PBS after aspirating the medium. Each well was then filled with 190 µL fresh TSB and 10 µL test compound, then incubated at 37°C in a stationary incubator. After 24 h incubation, the medium was carefully aspirated, and the wells washed twice with PBS, and stained with 100 µL resazurin dye (0.4% v/v from stock 1 g/100 ml). After 1 h incubation at 37°C, fluorescence was measured on a Flexstation: λex at 535 nm and λem at 590 nm. The percentage of surviving biofilm cells was calculated relative to the growth controls (Panda et al., 2020; Van Puyvelde et al., 2021).

### 2.4 Hydroquinone and benzoquinone quantification

A two-fold dilution series of the antibacterial compounds hydroquinone and benzoquinone was prepared, as well as a two-fold dilution series of hydroquinone in combination with an antioxidant (ascorbic acid or glutathione) and a two-fold dilution series of the individual antioxidants, in a 96–well V-shaped bottom polystyrene microtiter plate. Of each step in the dilution series, ten μL was added to the wells of the 96-well flat-bottom plate (Corning, UV transparent flat bottom) containing the 190 μL TSB medium with bacteria. The plate was then immediately (0 hours’ time point) placed in an Infinite M200 spectrophotometer, and the absorbance was measured from 320 to 620 nm in 5 nm increments. After incubation at 37°C (24-hour time point), a second, identical absorbance measurement was performed. As a control, a medium without bacterial cells was used to check for contamination and to calculate the absorbance by both hydroquinone and benzoquinone (due to a red coloration caused by the compounds).

The absorbance of hydroquinone, benzoquinone, hydroquinone with ascorbic acid, and hydroquinone with glutathione was measured at time points of 0 hours and 24 hours. Absorbance at 0 hours, absorbance at 24 hours, and concentration at 0 hours are known values; concentration at 24 hours is unknown. To calculate gain or loss of benzoquinone, absorption measurements at 350 nm were used since benzoquinone shows a clear absorption peak at 350 nm, while hydroquinone does not.

A calibration curve was established by plotting the absorbance at 0 hours (Y-axis) against the concentration at 0 hours (X-axis) and fitting a straight line through it. This calibration curve provides an equation y = mx + b; with m being the slope, and b the value of the intercept with the Y-axis. The following formula was then used to calculate the concentration at 24 hours:


$$\text{Concentration at }24\text{ hours}=\frac{\text{Absorbance at }24\text{ hours }-\text{ b}}{\text{m}}$$

### 2.5 Statistical analyses

All data were analyzed using GraphPad Prism 6.0 software (San Diego, CA). All bioassays were independently repeated at least twice, each time with duplicate technical repeats. Error bars represent standard deviations unless otherwise stated. Dose-response curves were analyzed with GraphPad Prism 6.0 Software to estimate half maximal inhibitory concentrations (IC_50_) using a non-linear least-squares sigmoid regression. More specifically, a log(inhibitor) *vs*. response curve with a fixed top value of 100%, a fixed bottom value of 0%, and a fixed Hill slope of 1 was used (Kerkoub et al., 2018).

## 3 Results

### 3.1 Isolation and identification of hydroquinone from *C. ferruginea* leaf

Previously, we studied the antimicrobial compounds in extracts of *C. ferruginea* leaves through bioassay-guided purification using *Staphylococcus aureus* (planktonic) as a target organism (Kouakou et al., 2019). Two major compounds were identified using a combination of NMR and mass spectrometry: hydroquinone and caffeic acid methyl ester (**Supplementary Materials**, **Supplementary Figure S3**). Hydroquinone exhibits clear antibacterial activity (IC_50_ = 63 μg/mL) (Kouakou et al., 2019). However, it was noticed that during incubation for antimicrobial testing, the color of the medium in the 96-well plate changed from colorless to dark red, probably due to the oxidation of hydroquinone into benzoquinone. To know which of the two compounds has a stronger effect on microbes, we tested both hydroquinone and benzoquinone in different concentrations against *S. aureus* strain USA300 and *S. aureus* subsp. *aureus* Rosenbach using a broth dilution method. In addition, we tested both compounds (at pH ±7.0) after addition of two antioxidants: ascorbic acid and glutathione.

### 3.2 Oxidation inhibition and anti-staphylococal activity (planktonic)

**Figure 1:**
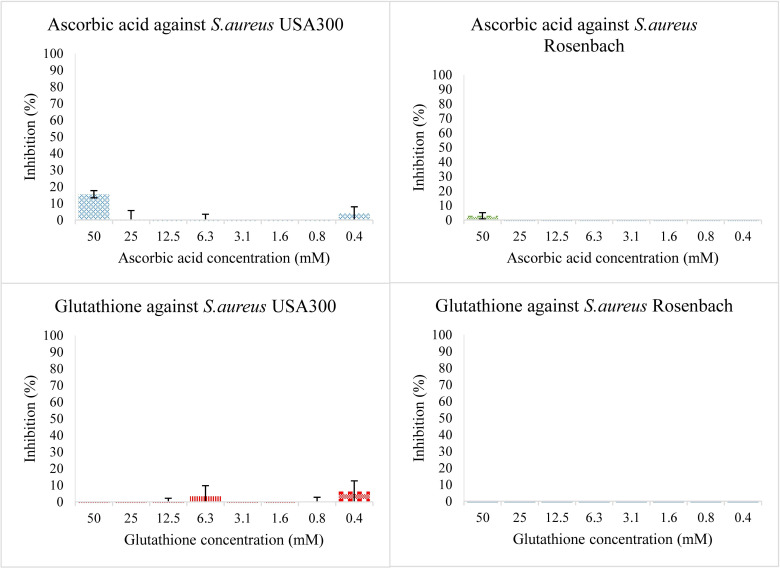
Bacterial growth in the presence of two-fold dilution series of the antioxidants ascorbic acid (top) and glutathione (bottom) in TSB medium pH 7.0. The X-axis shows the concentration of the added compound, the Y-axis shows the % bacterial inhibition compared to the solvent control. The error bars show the range of values in all data sets used; the top cap is the maximum value, and the bottom cap is the minimum value. The graphs on the left show inhibition percentage of the compound against *S. aureus* USA 300 (planktonic) and the ones on the right against *S. aureus* Rosenbach (planktonic).

**Figure 2:**
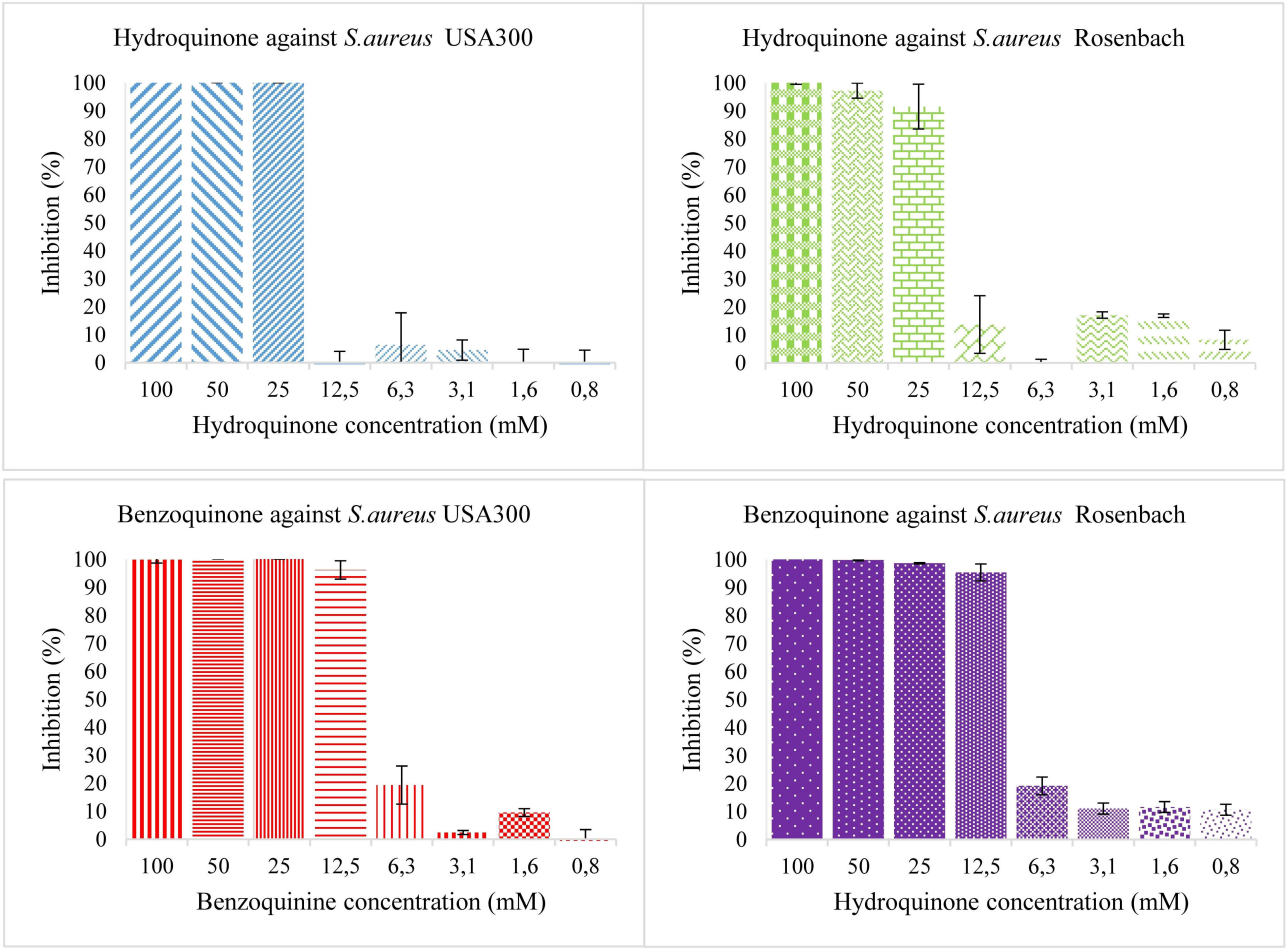
Bacterial growth in the presence of two-fold dilution series of hydroquinone (top) and benzoquinone (bottom) in TSB medium pH 7.0. X-axis shows the concentration of the added compound and Y-axis shows the % growth inhibition compared to solvent control. The error bars show the range of values in all data sets used; the top cap is the maximum value, and the bottom cap is the minimum value. The left panels show the results for *S. aureus* USA 300 (planktonic), the right ones for *S. aureus* Rosenbach (planktonic).

Individual antioxidants were tested for antibacterial activity at different concentrations, starting from 50 mM and diluting to 25, 12.5, 6.25, 3.12, and 1.56 mM. Neither antioxidant shows significant antibacterial activity (**Figure 1**). Both hydroquinone and benzoquinone were tested at similar concentrations. In isolation (without antioxidants), both compounds are effective at inhibiting both bacterial strains (**Figure 2**). For *S. aureus* USA 300 (planktonic), hydroquinone showed an IC_50_ value of 19.2 ± 4.8 mM, while benzoquinone showed an IC_50_ value of 7.7 ± 2.7 mM. For *S. aureus* Rosenbach, the corresponding IC_50_ values were similar: 15.6 ± 2.6 mM, and 6.9 ± 2.3 mM, respectively.

**Figure 3:**
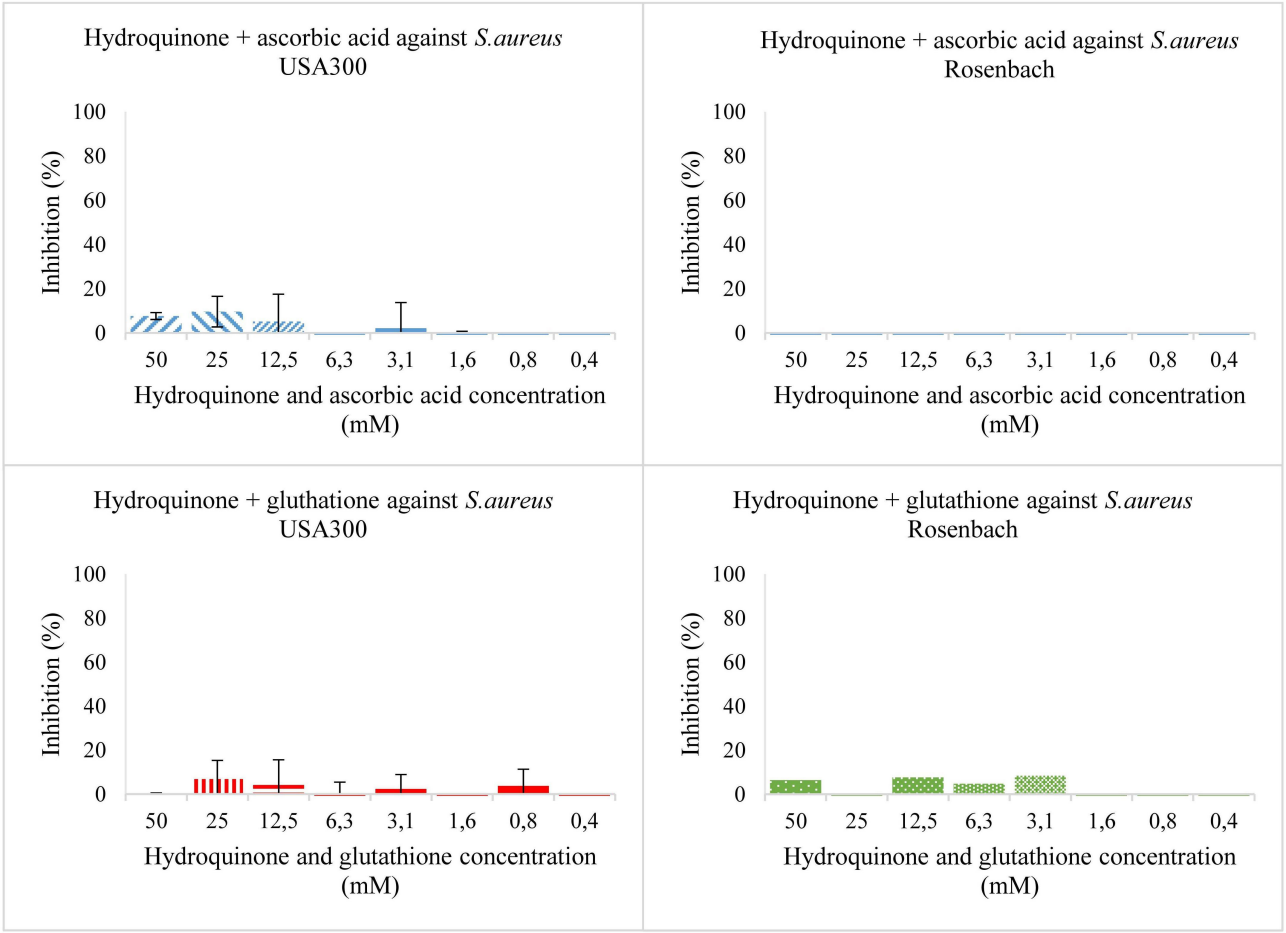
Bacterial growth in the presence of two-fold dilution series of hydroquinone with ascorbic acid (top) and hydroquinone with glutathione (bottom) in TSB medium pH 7.0. X-axis shows the concentration of the added compound, and Y-axis the % growth inhibition compared to solvent. The error bars show the range of values in all data sets used; the top cap is the maximum value, and the bottom cap is the minimum value. The left panels show results for *S. aureus* USA 300 (planktonic), the right ones for *S. aureus* Rosenbach (planktonic).

Then, using the same concentrations of both compounds, an antioxidant was added (**Figure 3**). For *S. aureus* USA 300, as well as *S. aureus* Rosenbach, hydroquinone combined with ascorbic acid or glutathione, showed no or only slight antibacterial activity so an IC_50_ value could not be estimated. These experiments were labelled condition two and three for ascorbic acid and glutathione, respectively. They imply that hydroquinone has no antibacterial effect at the concentrations tested, and that the antibacterial effects of hydroquinone observed in earlier experiments are largely (if not entirely) due to its conversion into benzoquinone.

**Figure 4:**
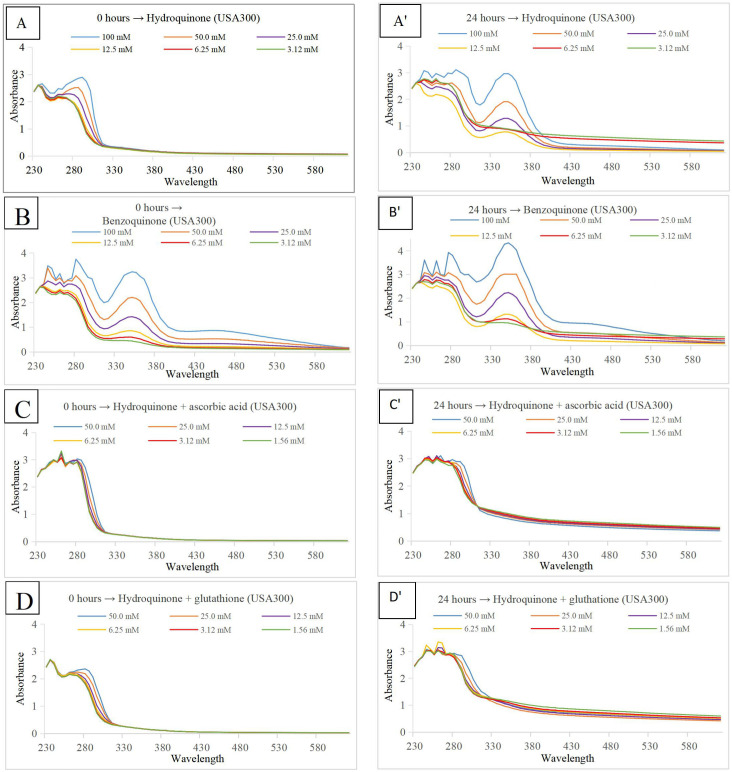
Absorbance (Y-axis) at wavelengths from 230 to 620 nm (X-axis). The color code at the bottom right in each panel indicates the concentration of either hydroquinone or benzoquinone, depending on the graph. *S. aureus* strain USA300 planktonic cells in TSB medium pH 7.0 were used for all experiments. Panels on the left show measurements at time point 0 hour, those on the right after an incubation period of 24 hours at 37°C. Compounds tested were hydroquinone **(A, A’)**, with benzoquinone **(B, B’)**, hydroquinone plus ascorbic acid **(C, C’)**, and hydroquinone plus glutathione **(D, D’)**.

To make sure only hydroquinone was present in conditions two and three (where ascorbic acid or glutathione were added, respectively), a multi-wavelength detector was used to measure Absorbance at wavelengths from 230 nm to 620 nm at two points in time. A first measurement was made directly after adding the compounds to the medium and/or bacterial cells (0-hour time point). A second measurement was made after an incubation at 37°C for 24 hours (24-hour time point). The absorbance values permit quantification of hydroquinone and/or benzoquinone, since hydroquinone only shows an absorbance peak around 290 nm, whereas benzoquinone shows absorbance peaks at 245 nm, 290 nm and 350 nm; the last peak is well separated from the hydroquinone peak (Padhi et al., 2020). Results for *S. aureus* strain USA300 can be found in **Figure 4**. Results for *S. aureus* subsp. *aureus* Rosenbach and medium without bacteria can be found in **Supplementary Materials** (**Supplementary Figure S3**).

### 3.3 Hydroquinone and benzoquinone quantification

Additional experiments were performed to estimate the amount of hydroquinone converted to benzoquinone. Results are presented in **Supplementary Materials** (**Supplementary Table S1**). As previously described, experiments were performed in the same conditions i.e. the first condition involving TSB medium (pH ±7.0) in which only hydroquinone or benzoquinone was added. The second and third condition involving the same TSB medium with hydroquinone and with the addition of antioxidants ascorbic acid (50 mM) or glutathione (50 mM), respectively. All conditions were tested in the absence of bacteria, in the presence of *S. aureus* subsp. *aureus* Rosenbach, and in the presence of *S. aureus* strain USA300 (planktonic).

### 3.4 Benzoquinone antibiofilm activity

Having established that benzoquinone was the active form, and given that it also showed activity on a biofilm-forming strain under planktonic growth conditions, we wanted to test whether it also showed activity against biofilms. This was indeed the case. Using the resazurin assay for biofilms formed by *S. aureus* USA 300, the pure benzoquinone exhibited an IC_50_ of 35.08 ± 8.23 mM.

## 4 Discussion

Hydroquinone as a reducing agent has a number of uses due to its high solubility in water. Most commonly used in photographic development solutions, such as dye or in the manufacture of rubber antioxidants (World Health Organization, International Programme on Chemical Safety, United Nations Environment Programme & International Labour Organization, 1996). Other industrial uses include as a stabilizer in paints, varnishes, motor fuels, and oils. As medicine for human use, hydroquinone (in concentrations up to 5%) is used to treat dyschromias (an alteration of the color of the skin or nails). The Food and Drug Administration (FDA) has approved one prescription product (Tri-Luma^®^) containing 4% hydroquinone along with other ingredients (0.01% luocinolone acetonide and 0.05% tretinoin).

As a depigmenting agent, it is used in a number of topical skin creams (up to 2%) as well as in other cosmetics, such as hair dyes and products for coating finger nails (World Health Organization, International Programme on Chemical Safety, United Nations Environment Programme & International Labour Organization, 1996). However, in 2006 the FDA proposed a ban on the use of hydroquinone in over-the-counter (OTC) skin products because it can cause permanent discoloration of the skin, and moreover is a suspected carcinogen. In plants (such as vegetables, fruits, grains, coffee beans, and tea leaves), it is present as free hydroquinone or as arbutin (hydroquinone β-D-glucopyranoside) (DeCaprio, 1999).

Over the past decade, the antibacterial properties of hydroquinone have been reported from several nature-derived sources. Kim et al. (2010) found antibacterial activity of several quinone derivatives, including hydroquinone, and suggested “that wheat germ extract has potential for the development of natural antimicrobials and food preservatives for controlling foodborne pathogens’’. Rúa et al. (2011) reported that hydroquinone was the strongest inhibitor among 19 phenolic compounds (including thymol, carvacrol, butylated hydroxyanisole, and gallic acid) that were tested against *S. aureus*, with IC_50_ values ranging from 62.5 to 100 μg/mL (Rúa et al., 2011).

Jyoti et al. (2016) used bioactivity-guided fractionation of an *Artemisia capillaris* extract active against *Mycobacterium tuberculosis* and isolated ursolic acid and hydroquinone as the major antimicrobial compounds with minimum inhibitory concentration (MIC) values of 12.5 μg/mL. Both compounds were also found to be effective at higher concentrations (MIC values of 12.5-25 μg/mL) against MDR/XDR *M*. *tuberculosis* strains (Jyoti et al., 2016). Zbikowska and co-workers studied antimicrobial and antiradical activity of five species of the genus *Bergenia*. They concluded that the most active antimicrobial substance is hydroquinone (Żbikowska et al., 2017). Jurica et al. (2017) studied the antimicrobial effect of the strawberry tree. They found arbutin as the most abundant bioactive compound in the leaves, whereas its metabolite hydroquinone was the principal factor responsible for the antibacterial activity. The strongest activity of hydroquinone was found against *Entercoccus faecalis* among 15 tested uropathogens, which was probably associated with the presence of bacterial β-glucosidases that convert arbutin to hydroquinone. Recently, Ma et al. (2019) investigated the antibacterial activity of *Ainsliaea bonatii*, and found that both hydroquinone and arbutin show strong antibacterial activity against *S. aureus*, methicillin-resistant *S. aureus* (MRSA), and extended spectrum β-lactamase *S. aureus*. Interestingly, the authors studied in detail the mechanism of action, and concluded that “hydroquinone could destroy the bacterial cell wall and membrane, increase permeability, lead to leakage of intracellular substance, affect synthesis of protein, and influence expression of genes.” In a previous study, Kouakou et al. (2019) used bioassay-guided purification to isolate antimicrobial compounds from the leaves of *C. ferruginea*. LC-MS and NMR revealed hydroquinone as the major compound responsible for antibacterial activity against *S. aureus*. Our hypothesis, however, suggests that the responsible compound is hydroquinone’s oxidation product: benzoquinone. Under mild conditions, hydroquinone undergoes oxidation into benzoquinone. This process can be reversed. It is clear that under normal (pH ±7.0) medium conditions, hydroquinone does oxidize to benzoquinone, no matter whether bacteria were present or not (**Figure 4**). This can be inferred from the absorbance peaks at 245, 290, and 350 nm, indicating the presence of benzoquinone (**Figures 4A, B**). When antioxidants ascorbic acid or glutathione were added (**Figures 4C, D**), there was no peak present at 350 nm, showing that oxidation was indeed inhibited; only hydroquinone was present, and benzoquinone was absent. In the presence of ascorbic acid (**Figure 4C**) or glutathione (**Figure 4D**) growth of microbial cells was not inhibited as strongly as in the first, neutral pH, condition when treated with either hydroquinone (**Figure 4A**) or benzoquinone (**Figure 4B**).

In the present study, the comparative analysis between planktonic and biofilm conditions for *S. aureus* revealed significant differences in their susceptibility to hydroquinone and benzoquinone. Under planktonic conditions, hydroquinone showed moderate antibacterial activity with IC_50_ values of 19.21 ± 4.84 mM for *S. aureus* USA 300 and 15.63 ± 2.62 mM for *S. aureus* Rosenbach, particularly when oxidation to benzoquinone was allowed. However, under conditions where hydroquinone oxidation was inhibited, growth inhibition was minimal, and reliable IC_50_ values could not be determined. In contrast, pure benzoquinone exhibited clear antibacterial activity, with IC_50_ values of 7.72 ± 2.73 mM and 6.90 ± 2.30 mM for the same strains, suggesting its potential utility against biofilm-forming *S. aureus* USA 300 under planktonic conditions. When examining biofilm conditions, the IC_50_ for benzoquinone against *S. aureus* USA 300 biofilms was significantly higher at 35.08 ± 8.23 mM, illustrating the well-known challenge that biofilms are generally more resistant to antimicrobial agents than their planktonic counterparts. This lower efficacy in biofilms may be due to the complex structure and protective environment they provide, making it difficult for antimicrobial compounds to penetrate and effectively eradicate the embedded bacteria. Additionally, hydroquinone was found inactive in this study, likely due to insufficient conversion to benzoquinone, reaffirming the need for oxidative conditions to achieve significant antibacterial effects. These findings underscore the importance of considering the distinct behaviors of planktonic and biofilm bacteria in the development of effective antimicrobial strategies.

Others have also described *S. aureus* antibiofilm activity of hydroquinones, presumably due to inhibition of virulence factor production (Kim et al., 2022). Hydroquinone itself was found inactive in this report (MIC > 400 µg/mL) (ibid.), presumably because it was not sufficiently converted into benzoquinone. Tert-butyl hydroquinone, a derivative of hydroquinone, is used as an antimicrobial food additive, with over 40 years of documented safe use. Its oxidation product (tert-butyl benzoquinone) is responsible for the antibacterial activity previously ascribed to tert-butylhydroquinone (Ooi et al., 2013). Tert-butylbenzoquinone causes loss of staphylococcal membrane integrity and is rapidly bactericidal. It displays potent activity against *S. aureus* biofilms *in vitro;* its ability to eradicate biofilms appears due to the fact that it can kill bacteria regardless of growth state Ooi et al., 2013). Recently, Carcamo-Noriega et al. (2019) identified 1,4-benzoquinone from scorpion venom and found remarkable antimicrobial activities against *S. aureus* and *Mycobacterium tuberculosis* compared with standard antibiotics. Structure-activity relationships of benzoquinones were studied by Omosa et al. (2016), and they conclude that “antibacterial activities gradually increased with increasing side chain length from 2, 3, 4 for **6, 7** and **8,** respectively, with optimal activities being realized with C7”. There is still more interest for organic chemists in exploring newer and interesting benzoquinone derivatives due to their potential applications.

Our results call into question previous work in which hydroquinone was claimed as the compound with antimicrobial activity against *S. aureus* or other microorganisms. They show that the presence of hydroquinone alone is not enough to inhibit microbial cell growth significantly and that benzoquinone shows more potential as an antimicrobial compound with possible pharmaceutical use. The previously reported antimicrobial activity of hydroquinone, however, can be accounted for by hydroquinone under physiological pH and oxygen conditions oxidizing (at least in part) into benzoquinone. It may be useful to also perform the antibacterial activity tests in the absence of oxygen. However, to carry out such experiments, specialized equipment such as an anaerobic chamber is needed. Moreover, under those conditions, only activity against microorganisms that grow under anaerobic conditions can be evaluated.

## 5 Conclusion

The present study demonstrates that benzoquinone is formed due to extensive conversion of hydroquinone during the course of a 24-hour incubation period, typical of many antimicrobial assays, and that the former probably accounts for most (if not all) of the (reported) antimicrobial activity of the latter, which acts like a prodrug. The bioactivity of hydroquinone, therefore, will depend on conditions favoring this conversion and may not materialize when the conversion is inhibited. At ambient temperatures and in the presence of oxygen, the conversion will be completed within hours, but anaerobic conditions or the presence of antioxidants may inhibit it. Such conditions unfavorable to conversion may obtain for instance in certain packaged foods. Earlier reports on the antimicrobial activity of hydroquinone may need to be re-evaluated in light of our results.

## Data Availability

The original contributions presented in the study are included in the article/**Supplementary Material**. Further inquiries can be directed to the corresponding author.
