# Supplementary material for: The anti-staphylococcal activity (planktonic and biofilm) of Cnestis ferruginea is due to benzoquinone, the oxidation product of hydroquinone
Source: Front Cell Infect Microbiol. 2025 Jan 17;14:1494589. doi: 10.3389/fcimb.2024.1494589 (PMC11782209; doi:10.3389/fcimb.2024.1494589)
Supplement: Supplementary file 1 [file DataSheet1.docx]

**The anti-staphylococcal activity (planktonic and biofilm) of *Cnestis ferruginea* is due to benzoquinone, the oxidation product of hydroquinone**

Sujogya Kumar Panda^1,2†*^, Michelle Reynders^1†^, Purity N. Kipanga^1^ and Walter Luyten^1^

***** Correspondence: sujogyapanda@soa.ac.in (S.K.P.)

**Supplementary material(s)**

**Supplementary Figure 1.** : Extraction and separation of phytochemicals for bioassay-guided fractionation (Kouakou et al., 2019)

| 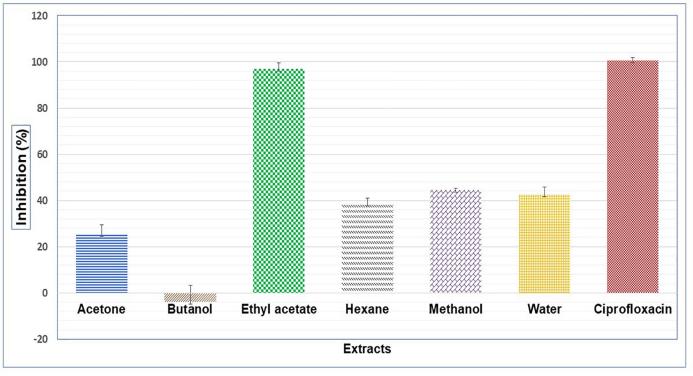 | 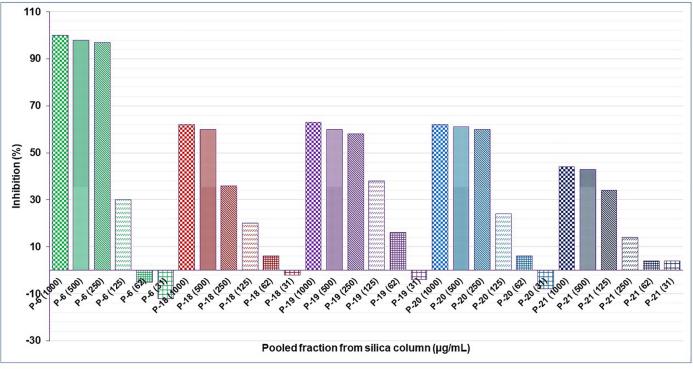 |
| --- | --- |
| **Supplementary Figure 2(a)** : Antibacterial activity on *S. aureus* of different extracts prepared with liquid-liquid separation of an aqueous extracts of *C. ferruginea* leaf; positive control ciprofloxacin (Kouakou et al., 2019). | **Supplementary Figure 2(b)** : Anti-staphylococcal activity of serial dilution of the active pooled fractions from a silica gel column of the EtOAc subfraction of an aqueous extract of *C. ferruginea* (Kouakou et al., 2019)*.* |


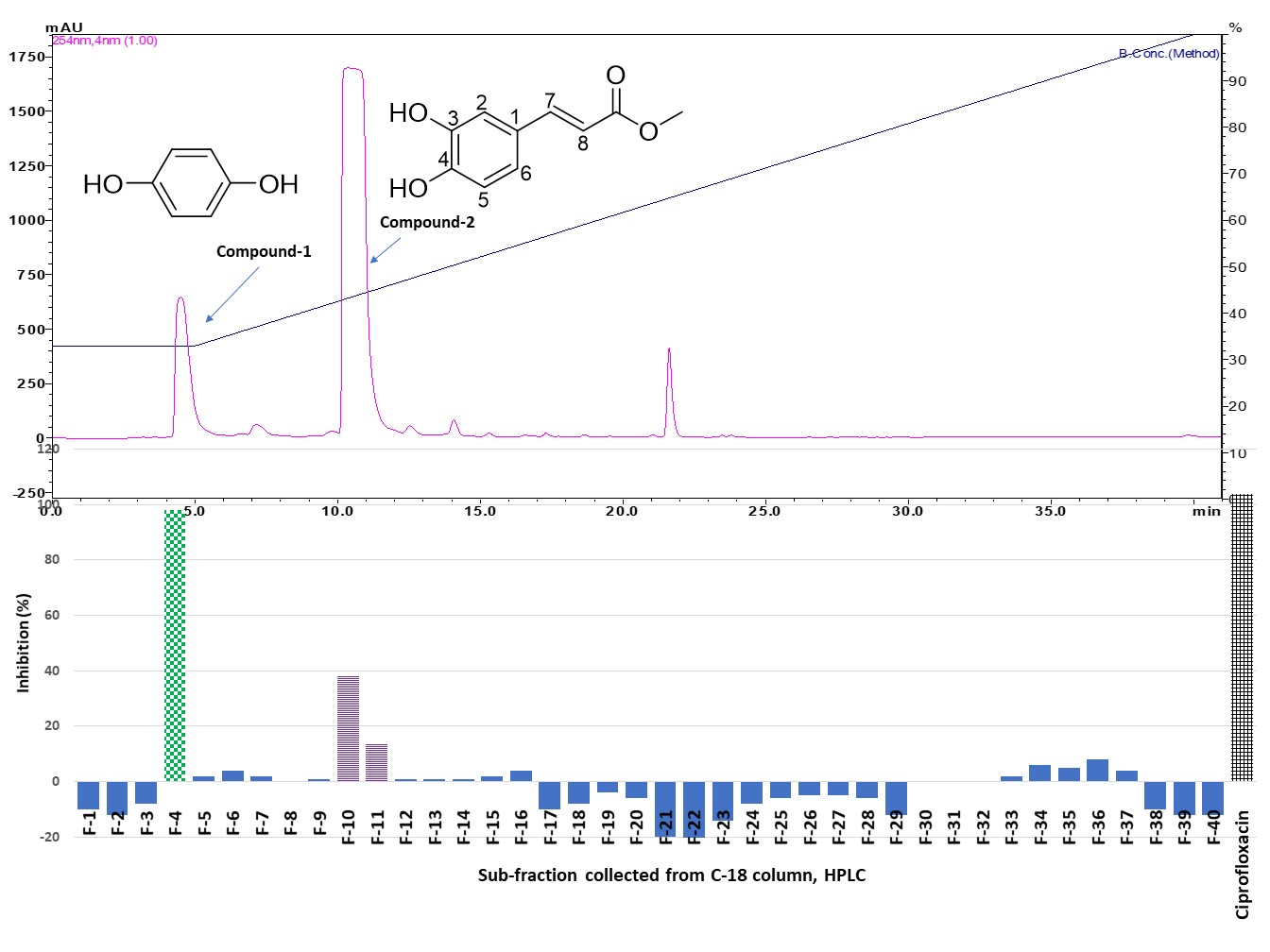


**Supplementary Figure 3.** Chromatogram of pooled fraction 6 (P6) of *Cnestis ferruginea* with reversed-phase HPLC on a C18 column. The first peak is responsible for most of the antimicrobial activity and was identified as hydroquinone through NMR and LC-MS. Peak 2 was identified as caffeic acid methyl ester, which has moderate activity. For more details, see Kouakou *et al*. (2019).


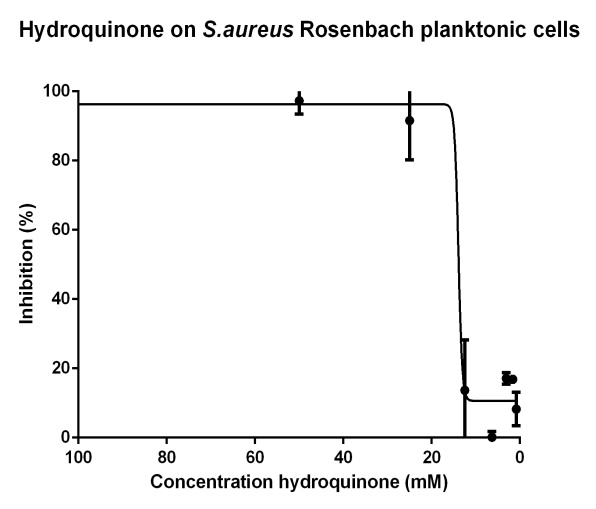

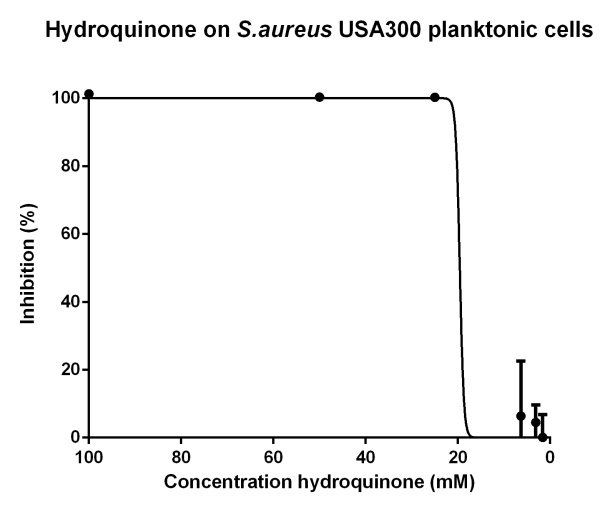


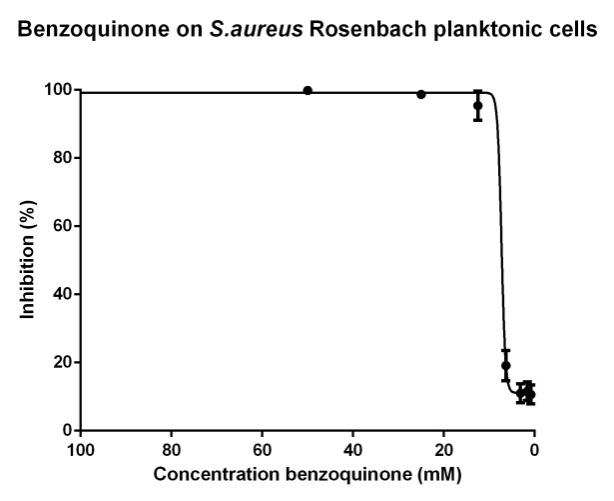

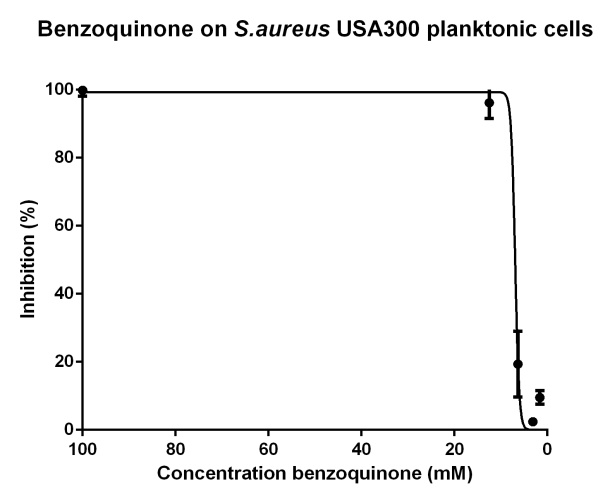


**Supplementary Figure 4: Dose-response curves of two-fold dilution series of hydroquinone (top) and benzoquinone (bottom) in TSB medium pH 7.0.** The X-axis shows the concentration of the added compound, and the Y-axis shows the corresponding bacterial inhibition. The panels on the left are for *S. aureus* USA 300, those on the right for *S. aureus* Rosenbach.

**A**

**B**

**Supplementary Figure 5:**

**Absorbance (Y-axis) at wavelengths from 230 to 620 nm (X-axis).** The colour code at the bottom right in each panel indicates the initial concentration of either hydroquinone or benzoquinone, depending on the graph. *S. aureus* Rosenbach planktonic cells in TSB medium pH 7.0 were used for experiments A-D: for E-H only TSB medium was used without bacteria. Panels on the left show measurements at time point 0 hour, those on the right after an incubation period of 24 hours at 37°C. Compounds tested were: hydroquinone (A, A’, E, E’), with benzoquinone (B, B’, F, F’), hydroquinone plus ascorbic acid (C, C’, G, G’) and hydroquinone plus glutathione (D, D’, H, H’).

**Supplementary material Table 1:**

Amount of hydroquinone converted to benzoquinone after 24 hours. Concentration of benzoquinone estimated by measuring absorbance at 350 nm. Four starting conditions: addition of hydroquinone (1Hydro), benzoquinone (1Benzo), hydroquinone with ascorbic acid and (2) or hydroquinone with glutathione (3) to the medium. Three media conditions: no bacterial cells (A), *S. aureus* subsp. *aureus* Rosenbach (B) or *S. aureus* strain USA300 (C).

|  | Concentration  at 0 hours (mM) | Concentration  at 24 hours (mM) | Benzoquinone gain/loss (mM) |
| --- | --- | --- | --- |
| Condition 1HydroA  - No antioxidants  - No bacteria | \| 3.12 \| \| --- \| \| 6.25 \| \| 12.5 \| \| 25 \| \| 50 \| \| 100 \| \|  \| | \| 5432.0 \| \| --- \| \| 3544.6 \| \| 2123.8 \| \| 1116.6 \| \| 562.00 \| \| 467.20 \| | \| 5428.9 \| \| --- \| \| 3538.3 \| \| 2111.3 \| \| 1091.6 \| \| 512.00 \| \| 367.20 \| |
| Condition 1HydroB  - No antioxidants  - Rosenbach | \| 3.12 \| \| --- \| \| 6.25 \| \| 12.5 \| \| 25 \| \| 50 \| \| 100 \| \|  \| | \| 1373 \| \| --- \| \| 1354.8 \| \| 1441.6 \| \| 2032.6 \| \| 3776.8 \| \| 5668.8 \| | \| 1369.9 \| \| --- \| \| 1348.6 \| \| 1429.1 \| \| 2007.6 \| \| 3726.8 \| \| 5568.8 \| |
| Condition 1HydroC  - No antioxidants  - USA300 | \| 3.12 \| \| --- \| \| 6.25 \| \| 12.5 \| \| 25 \| \| 50 \| \| 100 \| | \| 1307.6 \| \| --- \| \| 1272.4 \| \| 1073.0 \| \| 2096.6 \| \| 3354.8 \| \| 5475.4 \| | \| 1304.5 \| \| --- \| \| 1266.2 \| \| 1060.5 \| \| 2071.6 \| \| 3304.8 \| \| 5375.4 \| |
| Condition 2A  - Ascorbic acid  - No bacteria | \| 1.56 \| \| --- \| \| 3.12 \| \| 6.25 \| \| 12.5 \| \| 25 \| \| 50 \| | \| -382 \| \| --- \| \| -603 \| \| -844 \| \| -1238 \| \| -2143 \| \| -4417 \| | \| -383.60 \| \| --- \| \| -606.10 \| \| -850.30 \| \| -1250.5 \| \| -2168.0 \| \| -4467.0 \| |
| Condition 2B  - Ascorbic acid  - Rosenbach | \| 1.56 \| \| --- \| \| 3.12 \| \| 6.25 \| \| 12.5 \| \| 25 \| \| 50 \| | \| 8871.1 \| \| --- \| \| 8628.9 \| \| 8786.7 \| \| 8550.0 \| \| 7847.8 \| \| 7783.3 \| | \| 8869.6 \| \| --- \| \| 8625.8 \| \| 8780.4 \| \| 8537.5 \| \| 7822.8 \| \| 7733.3 \| |
| Condition 2C  - Ascorbic acid  - USA300 | \| 1.56 \| \| --- \| \| 3.12 \| \| 6.25 \| \| 12.5 \| \| 25 \| \| 50 \| | \| -16364 \| \| --- \| \| -15498 \| \| -15338 \| \| -14608 \| \| -13932 \| \| -12482 \| | \| -16365.6 \| \| --- \| \| -15501.1 \| \| -15344.3 \| \| -14620.5 \| \| -13957.0 \| \| -12532.0 \| |
| Condition 3A  - Glutathione  - No bacteria | \| 1.56 \| \| --- \| \| 3.12 \| \| 6.25 \| \| 12.5 \| \| 25 \| \| 50 \| | \| 2355.5 \| \| --- \| \| 1321.5 \| \| 2442.0 \| \| 3780.5 \| \| 5738.0 \| \| 6766.5 \| | \| \| 2353.9 \| \| --- \| \| 1318.4 \| \| 2435.8 \| \| 3768.0 \| \| 5713.0 \| \| 6716.5 \| \| \| --- \| --- \| --- \| --- \| --- \| --- \| --- \| |
| Condition 3B  - Glutathione  - Rosenbach | \| 1.56 \| \| --- \| \| 3.12 \| \| 6.25 \| \| 12.5 \| \| 25 \| \| 50 \| | \| 12901.4 \| \| --- \| \| 13870.0 \| \| 13707.1 \| \| 13094.3 \| \| 12538.6 \| \| 12020.0 \| | \| 12899.9 \| \| --- \| \| 13866.9 \| \| 13700.9 \| \| 13081.8 \| \| 12513.6 \| \| 11970.0 \| |
| Condition 3C  - Glutathione  - USA300 | \| 1.56 \| \| --- \| \| 3.12 \| \| 6.25 \| \| 12.5 \| \| 25 \| \| 50 \| | \| -19320 \| \| --- \| \| -18122 \| \| -18218 \| \| -16586 \| \| -14804 \| \| -16984 \| | \| -19321.6 \| \| --- \| \| -18125.1 \| \| -18224.3 \| \| -16598.5 \| \| -14829.0 \| \| -17034.0 \| |
| Condition 1BenzoA  - No antioxidants  - No bacteria | \| 3.12 \| \| --- \| \| 6.25 \| \| 12.5 \| \| 25 \| \| 50 \| \| 100 \| | \| 6.400 \| \| --- \| \| 16.20 \| \| 27.70 \| \| 52.80 \| \| 85.10 \| \| 120.5 \| | \| 3.30 \| \| --- \| \| 10.0 \| \| 15.2 \| \| 27.8 \| \| 35.1 \| \| 20.5 \| |
| Condition 1BenzoB - No antioxidants  - Rosenbach | \| 3.12 \| \| --- \| \| 6.25 \| \| 12.5 \| \| 25 \| \| 50 \| \| 100 \| | \| 18.90 \| \| --- \| \| 19.80 \| \| 27.30 \| \| 49.40 \| \| 79.40 \| \| 119.6 \| \|  \| | \| 15.8 \| \| --- \| \| 13.5 \| \| 14.8 \| \| 24.4 \| \| 29.4 \| \| 19.6 \| |
| Condition 1BenzoC - No antioxidants  - USA300 | \| 3.12 \| \| --- \| \| 6.25 \| \| 12.5 \| \| 25 \| \| 50 \| \| 100 \| | \| 1307.6 \| \| --- \| \| 1272.4 \| \| 1073.0 \| \| 2096.6 \| \| 3354.8 \| \| 5475.4 \| | \| 1304.5 \| \| --- \| \| 1266.2 \| \| 1060.5 \| \| 2071.6 \| \| 3304.8 \| \| 5375.4 \| |
